# The Segmental Morphometric Properties of the Horse Cervical Spinal Cord: A Study of Cadaver

**DOI:** 10.1155/2013/734923

**Published:** 2013-02-07

**Authors:** Sadullah Bahar, Durmus Bolat, Muhammet Lutfi Selcuk

**Affiliations:** ^1^Department of Anatomy, College of Veterinary Medicine, University of Selcuk, Selcuklu, 42075 Konya, Turkey; ^2^Department of Anatomy, College of Veterinary Medicine, University of Kirikkale, Yahsihan, 71451 Kirikkale, Turkey

## Abstract

Although the cervical spinal cord (CSC) of the horse has particular importance in diseases of CNS, there is very little information about its segmental morphometry. The objective of the present study was to determine the morphometric features of the CSC segments in the horse and possible relationships among the morphometric features. The segmented CSC from five mature animals was used. Length, weight, diameter, and volume measurements of the segments were performed macroscopically. Lengths and diameters of segments were measured histologically, and area and volume measurements were performed using stereological methods. The length, weight, and volume of the CSC were 61.6 ± 3.2 cm, 107.2 ± 10.4 g, and 95.5 ± 8.3 cm^3^, respectively. The length of the segments was increased from *C*_1_ to *C*_3_, while it decreased from *C*_3_ to *C*_8_. The gross section (GS), white matter (WM), grey matter (GM), dorsal horn (DH), and ventral horn (VH) had the largest cross-section areas at *C*_8_. The highest volume was found for the total segment and WM at *C*_4_, GM, DH, and VH at *C*_7_, and the central canal (CC) at *C*_3_. The data obtained not only contribute to the knowledge of the normal anatomy of the CSC but may also provide reference data for veterinary pathologists and clinicians.

## 1. Introduction

The spinal cord (SC) is macroscopically or microscopically affected by aging and disease, like the other organs. To determine or monitor these alterations, using quantitative data is more effective than using qualitative evaluation. The morphometric differences, in terms of length, diameter, area, and volume, of SC segments between humans and other animal species have been revealed in previous research. These studies can be divided into three groups according to their methodology: (a) macroscopic [1, 2], (b) microscopic [3, 4], and (c) medical imaging [5–7]. In the last group, measurements have mostly been performed by manual segmentation or by taking into account the vertebrae [6, 8, 9]. In the first two groups, studies in which segmentation has been performed, measurements of the diameter and cross-sectional area have been performed on a certain region of the segments (a) or on a section taken from a certain region of tissue (b).

Stereological methods that depend on the effective sampling of biological tissues have been performed frequently since 1980 for effective calculation of volume, surface area, length, and number parameters of tissue without bias [10, 11]. These techniques are used, especially in the brain, to calculate the volume, cortical surface area, and the number of cells [12, 13]. Limited information on segmental morphometry obtained using the techniques mentioned has been provided by research performed on the SC of rats [14], mice [14, 15], and chickens [4]. 

Diseases of the cervical spinal cord (CSC) have a special importance among the central nervous system diseases of the horse because of their high prevalence, clinical signs and often poor prognosis [16, 17]. In addition to supportive diagnostic radiography, medical imaging methods (Magnetic Resonance Imaging and Computerized Tomography) have been used in recent years to diagnose and monitor disease progress [8]. Morphometric evaluations of the SC and its surrounding tissue are often used in medical imaging, as in histopathological studies [6, 8, 18–20]. Unlike many other mammalian species including humans, the morphometric data obtained postmortem from horses and interpreted as reference values have been realized to be quite inadequate.

The objectives of this study were to determine the morphometric features of the CSC of mature horses macroscopically and to reveal the microscopic morphometry using stereological methods. The data obtained were used to investigate possible relationships among the compartments of the SC.

## 2. Materials and Methods

### 2.1. Animals

The present study was performed on the SC of five horses of different breeds and sex (a 12-year-old male Thoroughbred weighing 450 kg, a 10-year-old male Thoroughbred weighing 420 kg, a 15-year-old male Belgian horse weighing 480 kg, a 13-year-old female Arabian horse weighing 300 kg, and a 15-year-old female Shetland pony weighing 230 kg) that were scheduled for euthanasia upon being diagnosed with various orthopedic disorders after referral to the Equestrian Facilities of Selcuk University, Faculty of Veterinary Medicine. The study protocol was approved by the Ethic Committee. The animals were anaesthetized by administration of 10% chloral hydrate (80 mg/kg, I.V.) [21] and killed under general anesthesia afterwards; 10% neutral formalin solution was perfused via the common carotid artery.

### 2.2. Dissection, Extraction, and Segmentation

Dissection was performed ten days after the fixation procedure. The brain was opened with a dorsal approach, and the SC was exposed by laminectomy. The SC was cut transversally in front of the roots of the *C*_1_ spinal nerve, preserving the dura mater, and removed from the brainstem. The spinal dura mater and arachnoid mater were opened dorsally. The border between the two segments was determined as described previously [2]. Segmentation of the SC was completed by cutting transversally from the indicated points, except for the caudal segments.

### 2.3. Macroscopic Measurements

The length and diameter of each segment was measured using digital calipers, and segment volume was assessed by Archimedes' principle. All segments were weighed with an assay balance (0.01 g). Only weight and volume measurements were performed on the brain.

### 2.4. Stereological Design and Microscopic Analysis

Volume parameters were utilized using Cavalieri's principle. The following steps were included in this method: tissue sampling, determination of surface area, and volume calculation [10, 22, 23].

**Figure 1:**
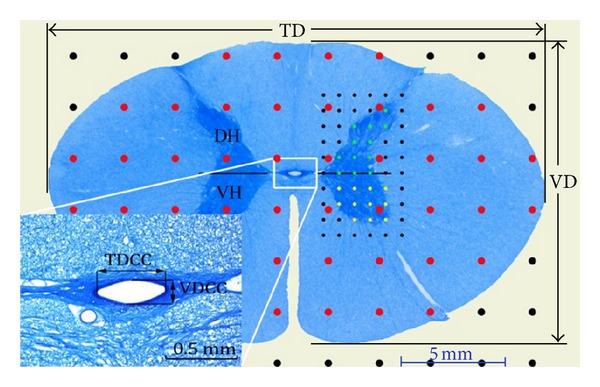
Gross-section of the spinal cord (scanned image), central canal (microscopic image), and measurements of area (point counting method) and diameter performed on image. Transversal diameter (TD), vertical diameter (VD), dorsal horn (DH), ventral horn (VH), transversal diameter of central canal (TDCC), vertical diameter of central canal (VDCC). Red points are used to calculate the area of gross section, green points are used to measure the area of dorsal horn, and yellow points are used to calculate the area of ventral horn.

The segments were sampled in accordance with a systematic random sampling principle before the histological procedures [10, 24]. For this procedure, a tissue slicer was prepared with microtome blades that were placed at 3.8 mm intervals, parallel to one another. The segments were placed perpendicular to the blades on the slicer so that the first tissue section was taken at random. All slabs belonging to the *C*_1_ and *C*_8_ segments were taken without sampling, while slabs taken from the other segments were sampled in 1/2 and 1/3 ratios. Slabs (8 or 13) were taken from the segments at the end of this procedure. All the slabs were numbered from a cranial to caudal direction and placed in trays, protecting their cranial cut surfaces. All the sections were dehydrated according to Bolat et al. [25], and paraffin blocks were prepared. One section from the first 30 consecutive sections taken from the paraffin block was obtained randomly using a rotary microtome at 10 *μ*m thickness and mounted on to a gelatinized glass slide. The sections were stored in a thermostatically controlled oven at 37°C for 24 hours and subsequently stained with modified May-Grunwald-Giemsa (Figure 1) [25]. The distance between two consecutive sections after the histological procedure was 3.4 mm for *C*_1_ and *C*_8_ segments, 6.8 mm for 1/2 percent sampled segments and 10.2 mm for 1/3 percent sampled segments.

The positive image scan option of a standard flatbed office scanner was used to obtain images for the measurements because the viewing area on the light and dissection microscope of the cross-section was large. Original-sized images (JPG, 600 DPI) of the sections were taken with this application (Figure 1). An imaging system adapted to the light microscope was used to obtain images to be used for measurement of the central canal (Figure 1). All images of the segments were kept separate by ordering them from the cranial to caudal direction on a PC for later analysis.

Measurements of VD and TD were performed on images taken from the GS and CC using ImageJ software (Figure 1). The mean value of the diameter obtained for each segment was recorded as the microscopic diameter value. The compression value of GS and CC was calculated using the following formula: VD/TD∗100. 

A point-counting grid was used to determine the area of the image of each section [10, 24]. ImageJ was calibrated first, and the grid function of the software was used to calculate the GS area of the cervical segments and its subcomponents. The area per point (*a*/*p*) was set for GS and WM at 6 mm^2^, for GM, DH, and VH at 0.6 mm^2^, and for CC at 3 *μ*m^2^. The grids were superimposed randomly on the section images (Figure 1). The total number of hits on each compartment of the SC was counted three times. The average of the total number of points was represented as  ∑*p*. The area and volume of each compartment of the SC were calculated using the formulas numbered (1) and (2), respectively. Consider

(1) $$\begin{array}{c} A_{\left(\text{mean}\right)}=\frac{\sum p*a\left(p\right)}{S_{n}}, \end{array}$$

(2) $$\begin{array}{c} V_{\text{est}}=\sum p*a\left(p\right)*t. \end{array}$$

*S*_*n*_ represents the number of tissue samples taken from a segment; *t* indicates the distance between two consecutive sections. Coefficient of error (CE) values were calculated according to Sahin and Ergur [26].

### 2.5. Statistical Analysis

The values were expressed as mean and standard error (mean ± SE). The diameters, areas, and volumes of cervical segments were compared using the Duncan test. The Pearson correlation test was also applied to investigate relationships among morphometric data values (SPSS 13.0). *P* < 0.05 was accepted statistically significant.

## 3. Results

The body weight of the animals used in the study, the weight of the fixed brain and SC, weight, length, and volume of the CSC, and the ratios and relative organ weight of the SC are given in Table 1.

### 3.1. Segment Lengths and Diameters

**Figure 2:**
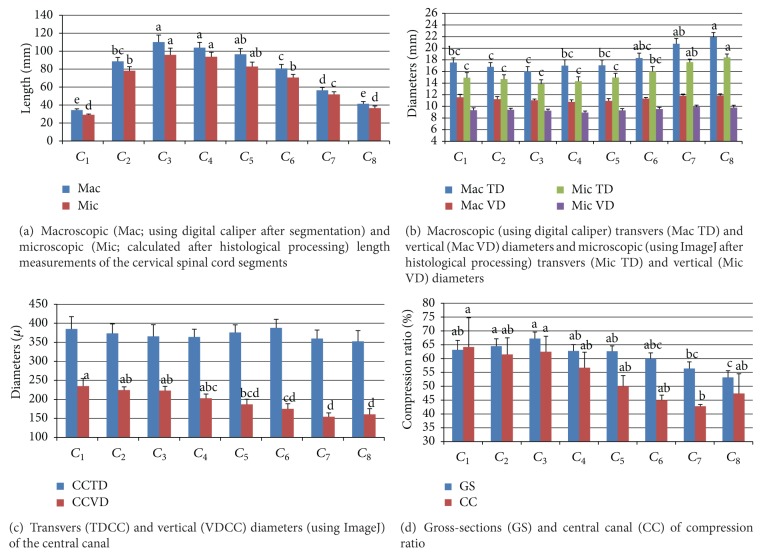
Length (a), diameters (b and c), and compression ratio (d) of cervical spinal cord segments. (a–e) Different letters on the top of columns of the same colour are statistically significant (Duncan test, *P* < 0.05, mean ± SE).

It was observed that segment length increased from *C*_1_ to *C*_3_ and decreased from *C*_3_ to *C*_8_ regularly, in both macroscopic and microscopic measurements (Figure 2(a)). The average 11.7% difference in the length of the CSC between measurements made by the two methods was caused by tissue shrinkage during the histological preparation. Segment diameters measured using digital calipers and the mean diameters of the GS of these segments are given in Figure 2(b). The shortest and longest TD of the SC segments was identified in *C*_3_ and *C*_8_, respectively. Although *C*_4_ had the shortest VD, there were no statistically significant differences among the VD values of the segments (Figure 1(b), *P* > 0.05). Tissue shrinkage for the TD and VD was determined 14.18% and 16.66%, respectively. Although *C*_6_ had the longest transverse diameter of CC (TDCC) (387.92 *μ*m), there were no statistically significant differences among the TDCC of the segments (Figure 2(c), *P* > 0.05). The longest and shortest vertical diameters of the CC (VDCCs) were seen in *C*_1_ and *C*_7_, and this diameter decreased regularly from *C*_1_ to *C*_7_ (Figure 2(c), *P* < 0.05). Compression ratios of GS and CC showed statistically significant differences, and this ratio decreased from *C*_1_ to *C*_8_ (Figure 2(d), *P* < 0.05). 

### 3.2. Cross-Sectional Areas of Segment Subcomponents

**Figure 3:**
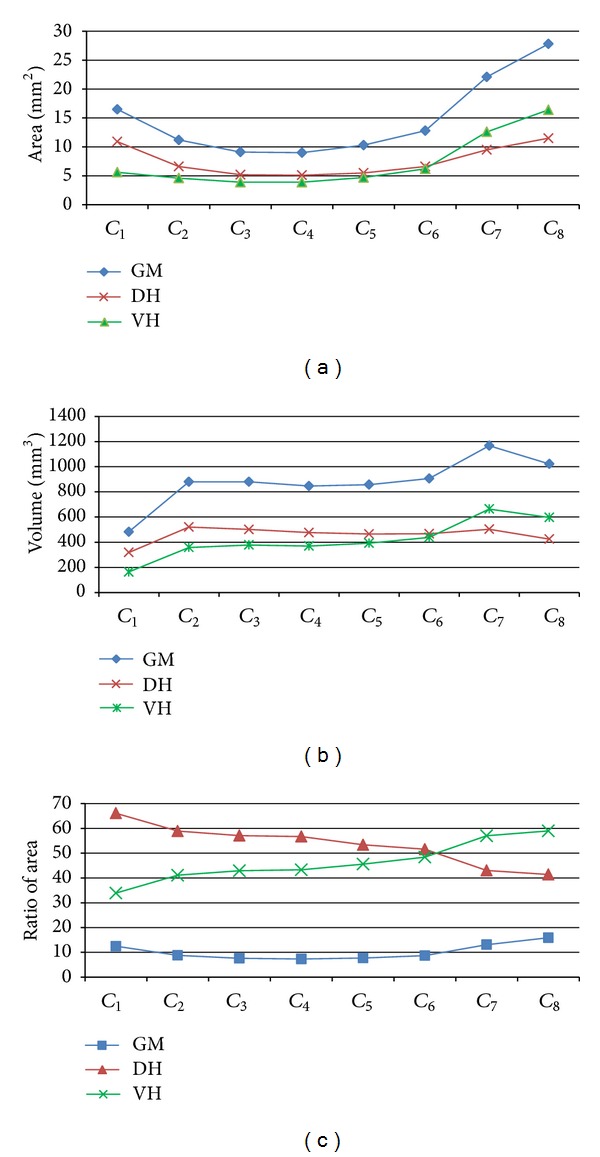
Grey matter (GM), dorsal horn (DH), and ventral horn (VH) of the segments, area (a), volume (b), and area ratio (GM/GS, DH/GM, and VH/GM) (c).

The mean areas of the segment subcomponents (GS, WM, GM, DH, VH, and CC) are given in Table 2, and the variation in the area and area ratio of GM, DH, and VH of the segments are represented in Figures 3(a) and 3(c). While the highest area values of GS, WM, and GM were determined in *C*_8_, the highest area value of CC was seen in *C*_3_ (Table 2). The area and area ratio of GM increased from *C*_4_ to *C*_8_, and VH contributed to this increment more than DH (Table 2 and Figures 3(a) and 3(c)).

### 3.3. Segment Volumes

Segment volumes measured by Archimedes' principle (before paraffin embedding) and the volumes of total segments and subcomponents (WM, GM, DH, VH, and CC) measured with Cavalieri's principle are given in Table 2. Variations in the volume of the GM, DH and VH of the segments are represented in Figure 3(b). Although *C*_4_ had the highest total segment volume and WM, *C*_2_ and *C*_6_ had similar volumes of total segment and WM statistically (*P* > 0.05, Table 2). The difference in the total segment volume (29.3%) between measurements made using Archimedes' and Cavalieri's principles was accepted as tissue shrinkage caused by histological processing.

### 3.4. Correlation Analysis

The results of the analysis of correlations among the morphometric parameters are given in Table 3. 

## 4. Discussion

The weight ratio of SC to the total weight of the central nervous system was 2% in the human, 6% in the gorilla, 23% in the dog, 30% in the cat, 40% in the horse, and 47% in the cow [27]. In the present study, we found the weight ratio to be 31.9% and the volume ratio to be 32.6% in the horse (Table 1).

In this study, lengths of CSC and SC were 61.2 and 167.2 cm, while a percent ratio of CSC/CS was 36.9% in the horse (Table 1). It has been reported that the lengths of CSC and SC in donkey [28], goat [29], brocket [30], mouse [15], and human [31] are 37.7–106.8 cm, 16.4–53.8 cm, 17.5–61.5 cm, 10.2–44 mm, and 9.4–43.1 cm, and percent ratios of CSC/CS of previously mentioned species are 35.3, 30.4, 28.5, 23.4, and 21.5, respectively. Barson and Sands [31] reported the weight of fresh SC and CSC of human to be 28.3–9.2 g (32.5%); these values were 249.6 and 107.2 g (42.8%) in the current study (Table 1). The length of the SC in domestic animals is directly proportional to the length of the vertebral column. The ratio of length and weight of the CSC of horse is greater than in other species, and this may be a result of the extended length of the cervical vertebral column in the horse when compared with other species. However, cervical segment length increased from *C*_1_ to *C*_3_ and decreased regularly from *C*_3_ to *C*_8_ in the horse, similar to the dog [32], donkey [33], and goat [29]. *C*_1_ was found to be the shortest segment, as reported in the dog, while *C*_8_ is reported to be the shortest segment in the donkey and goat (Figure 2(a)). The longest and the shortest segments were reported in sheep [34] and impala [35] to be *C*_2_ and *C*_8_, respectively.

The TD and VD of the segments are parameters that are used routinely in research, such as in postmortem [1, 3, 36] or medical imaging studies [5, 8, 37, 38], and as a morphometric or diagnostic tool for SC diseases. Measurements in medical imaging studies have been performed mostly by manual segmentation or by considering the vertebrae because it is difficult to differentiate the segment boundaries [8, 9, 18]. The longest and the shortest TD values have been reported for *C*_2_,  *C*_7_, and *C*_8_ of the horse [3], *C*_3_,  *C*_7_, and *C*_8_ of the donkey [28], *C*_2_ and *C*_6_ of the human (except *C*_1_) [36, 39], and  *C*_5_  and *C*_8_ (except *C*_1_ and *C*_2_) of the human [1], respectively, in previous research. In the current study, while TD decreased from *C*_1_ to *C*_3_, there was a regular increment from *C*_3_ to *C*_8_, and the highest TD value was found in *C*_8_ (Figure 2(b)). However, there was no statistically significant difference among the first five cervical segments in terms of TD (Figure 2(b), *P* > 0.05). Although the shortest and the longest VDs were found in *C*_4_ and *C*_8_, respectively, there was no statistically significant difference in VD (*P* > 0.05, Figure 2(b)). A similar situation has been observed in other studies conducted on the horse [3] and donkey [28]. However, the VD of human CSC is reported to decrease regularly from *C*_1_ to *C*_8_ in post-mortem histological studies [36, 39] and in antemortem studies using CT [40, 41] and MRI [7]. The ratio of the transverse diameter to the vertical diameter has been reported as the compression ratio in dogs [5] and humans [6, 36], and it has been used to evaluate pathological conditions. While this ratio gives simple information about the cross-sectional shape of the segments, it can also be used in comparisons among species. Thus, the compression ratio of the CSC of the horse (Figure 2(d)) in our study was very similar to compression ratios calculated using the results of a previous study conducted by Ocal and Haziroglu [28], whereas it was not similar to the compression ratios of the dog [5] and human [36]. VD can be used to determine lesion borders in lateral radiography, which is frequently preferred in the diagnosis of compressive disease of the CSC, and the compression ratio can be used as a morphometric parameter to diagnose pathological conditions of the CSC of the horse, like in dogs [5] and humans [6, 36, 42].

Occlusion of the central canal has been reported to start at 1-year-old human; after the fourth decade, the canal is completely occluded, except for the cervical segments, and in nine decades, the central canal of the entire SC is totally occluded [43, 44]. Although the diameter of the central canal in the dog [45] is known to be associated with ageing, no information about occlusion of central canal caused by ageing, except in association with pathological conditions, has been found in the literature. It could be said that the central canal of healthy domestic animals is more functional and stable than that of humans, and thus it has a specific morphometry. Morphometric characteristics of the central canal of the segments were examined in detail in the current research (Figures 2(c) and 2(d) and Table 2), and correlations between these morphometric data and other data from the segments were observed (Table 3). It was reported that the central canal was flattened dorsoventrally, especially in *C*_3_ and *C*_4_ of the horse [3], but in the donkey, it was flattened in *C*_1_ and between *C*_6_ and *C*_8_, it was rounded in *C*_2_ and *C*_5_, and it was flattened laterally in *C*_3_ and *C*_4_ [28]. In the current research, the shape of the central canal, which was flattened dorsoventrally, and the transverse diameter of the central canal was found to be similar to those reported in a previous study conducted by Braun [3] (however, the latter used only two animals), while the vertical diameter was different in the two studies. Although changes in the form of the central canal are seen in cross-sections taken from the affected area of the SC in congenital malformations and compressive disease of the cord, this subject has not been mentioned in detail in the literature [36, 46]. The central canal is a cerebrospinal fluid-filled space in the SC, and because it can be affected by pathological conditions, we think that it is appropriate to be taken into consideration in such situations. 

It has been reported that *C*_6_ (134 mm^2^) had the largest and *C*_2_ (86 mm^2^) had the smallest areas of GS; *C*_8_ had the largest (21.25 mm^2^) and *C*_2_ had the smallest areas of GM in the horse (*n* = 2) [3]. The largest and the smallest area of GS and GM were found in *C*_8_  (125 mm^2^) and *C*_3_  (89 mm^2^), *C*_8_  (25 mm^2^) and *C*_4_  (7 mm^2^). In humans these values were *C*_6_ (58.5 ± 7.2 mm^2^) and  *C*_8_  (51.2 ± 5.3 mm^2^), *C*_7_ (10.7 ± 1.3 mm^2^) and  *C*_3_ (7.2 ± 1.2 mm^2^), respectively (*n* = 12) [36]. The same writers reported that the value of GM increased from *C*_2_ (5.5%) in the horse, *C*_4_ (7.6%) in the donkey, and *C*_3_ (13.6%) in the human to *C*_8_ (17.6%, 19.84%, and 20.4%). Whereas *C*_3_ and  *C*_8_ had the largest and the smallest areas of GS, the area of grey matter decreased from *C*_1_ (55%) to *C*_8_ (32%) in a study conducted on rats [47]. In the current research, the largest and the smallest areas of GS and GM were detected at *C*_8_ and *C*_3_, *C*_8_ and *C*_4_ respectively. The GS areas of the first five segments were detected to be similar statistically, but the GM areas of only *C*_3_ and *C*_4_ were similar (Table 2, *P* > 0.05). The area of GM was determined to increase regularly from *C*_4_ (7.22%) to *C*_8_ (15.81%) (Figures 3(a) and 3(c)). Although our results were consistent with the results for the donkey [28], they were not compatible with the morphometric data for the horse reported by Braun [3]. Portiansky et al. [47] reported that morphometric differences between rats and other mammals are caused by the difference in the size and the number of neurons in the cord.

The morphological features of the DH and VH of cervical segments have been published in detail for the horse [3] and donkey [28]. These constituents of GM were evaluated morphometrically for the first time in the present study. It was seen that DH had the largest area in *C*_1_ and *C*_8_ and the smallest area in *C*_3_ and *C*_4_. VH had the largest area in *C*_8_ and the smallest area in *C*_2_ and *C*_4_. An inverse relationship was found between ratios in the ventral and dorsal horn, and at the *C*_6_ level, their areas, volumes, and ratios were almost equal (Table 2 and Figure 3).

Linear and volumetric changes in the brain have been investigated in detail after formalin fixation and paraffin embedding in previous research [48, 49]; however, the effect of these methods on the SC is not known. The transverse cross-sectional area was reduced by 13%-14% in the SC of rats [47], the human brain stem was reduced by 11%-12% transversally and 17% longitudinally [49], and the volumes of cerebral tissue [50], cerebral cortex, and WM [48] were decreased by 48%, 49%, and 58%, respectively, after paraffin embedding. In the current study, although the transverse cross-sectional area was not calculated before paraffin embedding, TD was reduced by 14.2%, VD by 16.66%, longitudinal shrinkage was 11.91%, and volumetric shrinkage of 29.3% was observed when the values for the diameters and volumes of the CSC determined before and after paraffin embedding were compared. Our results are compatible with previous results, except for longitudinal shrinkage, but there were differences in the materials and tissues. It is thought that the variation in volumetric shrinkage between cerebral tissue and the SC could be caused by the anatomical organization of tissues.

Positive correlations were reported between the TD and VD of CSC segments, and positive correlations were also reported among VD or GS areas and total segment volumes (*P* < 0.05) [1]. In the present study, a positive correlation was found between TD and VD or GS areas, a negative correlation between TD and total segment volume, and a positive correlation between the VD and DH and VH volumes, and there was no statistically significant difference between the GS area and total segment volume (Table 3).

## 5. Conclusion

The results of this study, in which detailed morphometric features of the CSC of mature horse were determined using stereological methods for the first time, will contribute to the knowledge of the related anatomical structures and can also be used as reference values by veterinary pathologists and clinicians for the quantitative evaluation of CSC disease.

## Figures and Tables

**Table 1:** The body weights of the animals used in the study and some morphometric measurements for the brains, cervical spinal cords, and spinal cords after neutral formalin fixation.

		Pony	Arabian	Belgium	TB I	TB II	Mean ± SE
	Body weight (kg)	230	300	480	420	450	376 ± 47.6

Brain	Weight (g)	455	490	570	590	555	532 ± 25.5
	Volume (cm^3^)	425	475	550	560	540	510 ± 25.9

SC	Weight (g)	207.2	186.3	305.9	267.0	281.6	249.6 ± 22.7
	Length (cm)	142.9	156.2	178.4	175.6	182.8	167.2 ± 7.6
	Volume (cm^3^)	204.7	180.3	297.1	270.9	283.7	247.3 ± 23.1

CSC	Weight (g)	87.8	77.3	130.8	116.6	123.4	107.2 ± 10.4
	Length(cm)	51.4	56.1	65.2	66.0	67.5	61.2 ± 3.2
	Volume (cm^3^)	77.4	73.5	111.7	104.1	110.8	95.5 ± 8.3

Ratio CSC/SC	Weight	42.4	41.5	42.8	43.7	43.8	42.8 ± 0.4
	Length	36.0	35.9	36.5	37.6	36.9	36.6 ± 0.3
	Volume	37.8	40.8	37.6	38.4	39.0	38.7 ± 0.6

RW	SC	0.090	0.062	0.064	0.064	0.063	0.068 ± 0.005
	CSC	0.038	0.026	0.027	0.028	0.027	0.029 ± 0.002

Spinal cord (SC), cervical spinal cord (CSC), relative weight (RW), and Thoroughbred (TB). Volume data were obtained by application of Archimedes' principle.

**Table 2:** Areas and volumes of subcomponents of the cervical spinal cord segments (mean ± SE, *n* = 5).

Area (mm^2^)							
Segment	GS	WM	GM	DH	VH	CC × 10^−3^	

*C*_1_	132 ± 9.9^c^	115 ± 9.8^b^	16.5 ± 0.7^c^	10.9 ± 0.7^a^	5.6 ± 0.2^cd^	58.9 ± 4.3^a^	
*C*_2_	127 ± 8.5^c^	115 ± 8.1^b^	11.2 ± 0.6^e^	6.6 ± 0.4^c^	4.6 ± 0.2^e^	58.1 ± 3.5^a^	
*C*_3_	120 ± 7.5^c^	111 ± 7.3^b^	9.1 ± 0.3^f ^	5.2 ± 0.2^d^	3.9 ± 0.2^e^	59.1 ± 5.7^a^	
*C*_4_	124 ± 8.6^c^	115 ± 8.3^b^	9.0 ± 0.3^f^	5.1 ± 0.2^d^	3.9 ± 0.3^e^	53.3 ± 2.6^a^	
*C*_5_	134 ± 8.7^c^	123 ± 8.2^ab^	10.3 ± 0.5^ef^	5.5 ± 0.3^cd^	4.7 ± 0.3^de^	50.9 ± 5.8^ab^	
*C*_6_	147 ± 10.4^bc^	134 ± 9.8^ab^	12.8 ± 0.6^d^	6.6 ± 0.3^c^	6.2 ± 0.4^c^	48.7 ± 6.1^ab^	
*C*_7_	169 ± 5.0^ab^	147 ± 5.3^a^	22.1 ± 0.4^b^	9.5 ± 0.3^b^	12.6 ± 0.4^b^	38.3 ± 4.8^b^	
*C*_8_	175 ± 8.0^a^	147 ± 7.7^a^	27.8 ± 0.6^a^	11.5 ± 0.5^a^	16.4 ± 0.4^a^	37.1 ± 2.8^b^	

Volume (*cm^3^, mm^3^)							

Segment	Arc*	Total	WM	GM	DH	VH	CC × 10^−3^

*C*_1_	5.7 ± 0.4^c^	3884 ± 387^c^	3402 ± 370^c^	483 ± 30^c^	319 ± 24^b^	164 ± 9^c^	1.7 ± 0.12^d^
*C*_2_	14.2 ± 1.5^ab^	10040 ± 1187^a^	9159 ± 1114^a^	880 ± 80^b^	521 ± 49^a^	359 ± 34^b^	4.5 ± 0.27^abc^
*C*_3_	17.1 ± 1.8^a^	11693 ± 1679^a^	10813 ± 1587^a^	880 ± 99^b^	502 ± 48^a^	378 ± 54^b^	5.6 ± 0.50^a^
*C*_4_	16.9 ± 1.8^a^	11731 ± 1313^a^	10884 ± 1247^a^	847 ± 66^b^	477 ± 36^a^	370 ± 34^b^	5.00 ± 0.33^ab^
*C*_5_	15.9 ± 1.8^ab^	11207 ± 1268^a^	10349 ± 1187^a^	858 ± 82^b^	465 ± 53^a^	392 ± 36^b^	4.2 ± 0.57^bc^
*C*_6_	14.6 ± 1.3^ab^	10387 ± 972^a^	9480 ± 915^a^	906 ± 59^b^	468 ± 36^a^	439 ± 28^b^	3.5 ± 0.58^c^
*C*_7_	11.7 ± 0.9^abc^	8956 ± 670^ab^	7790 ± 621^ab^	1167 ± 61^a^	503 ± 36^a^	664 ± 32^a^	2.1 ± 0.33^d^
*C*_8_	8.9 ± 1.0^bc^	6462 ± 5887^bc^	5441 ± 527^bc^	1022 ± 65^ab^	425 ± 41^ab^	597 ± 25^a^	1.4 ± 0.13^d^
Mean CE		0.044	0.046	0.046	0.052	0.054	0.053

Gross sections (GSs), white matter (WM), grey matter (GM), dorsal horn (DH), ventral horn (VH), central canal (CC). *Segment volumes calculated using Archimedes' principle after segmentation. Other volumetric measurements were obtained using the Cavalieri's principle. Coefficient of error mean (mean CE) was calculated according to Sahin and Ergur (2006) [26].

^a–f^: Different letters in the same column are statistically significant (Duncan test, *P* < 0.05).

**Table 3:** Relationships among microscopic measurements of the cervical spinal cords (*n* = 5).

		Length	Diameter				Area				Volume		
			TD	VD	TDCC	VDCC	GS	WM	GM	CC	Total	WM	GM
	CC	0.892***	−0.649***	−0.467**	0.003	0.279	−0.533***	−0.405**	−0.784***	0.643***	0.680***	0.702***	0.088
Volume	GM	0.312	0.195	0.141	−0.146	−0.251	0.660***	0.691***	0.348*	−0.419**	0.617***	0.573***	
	WM	0.911***	−0.431**	−0.128	−0.032	0.251	0.067	0.234	−0.506**	0.006	0.998***		
	Total	0.895***	−0.400*	−0.114	−0.04	0.224	0.108	0.271	−0.462**	−0.022			

	CC	0.265	−0.585***	−0.535***	0.028	0.262	−0.697***	−0.678***	−0.541***				
Area	GM	−0.747***	0.656***	0.312	−0.097	−0.409**	0.751***	0.603***					
	WM	−0.164	0.28	0.278	−0.054	−0.069	0.980***						
	GS	−0.324*	0.397*	0.309	−0.069	−0.16							

	VDCC	0.264	−0.545***	−0.115	−0.01								
Diameter	TDCC	−0.029	−0.132	−0.346*									
	VD	−0.265	0.589***										
	TD	−0.535***											

Transverse (TD) and vertical (VD) diameters of the segments, transverse (TDCC) and vertical (VDCC) diameters of central canal, gross section (GS), white matter (WM), grey matter (GM), and central canal (CC) area, spinal cord segments (total), and white matter (WM), grey matter (GM), and central canal (CC) volumes. Pearson's coefficient: *P < 0.05, **P < 0.01, and ***P < 0.001.
